# Characterization of oral microbiota and acetaldehyde production

**DOI:** 10.1080/20002297.2018.1492316

**Published:** 2018-07-06

**Authors:** Shigeyuki Yokoyama, Kenji Takeuchi, Yukie Shibata, Shinya Kageyama, Rie Matsumi, Toru Takeshita, Yoshihisa Yamashita

**Affiliations:** a Section of Preventive and Public Health Dentistry, Division of Oral Health, Growth and Development, Faculty of Dental Science, Kyushu University, Fukuoka, Japan; b OBT Research Center, Faculty of Dental Science, Kyushu University, Fukuoka, Japan

**Keywords:** Alcohol, bacteria, oral cancer, oral cavity, pyrosequencing, saliva

## Abstract

**Background**: *Neisseria* has been reported to be a high producer of acetaldehyde (ACH), a carcinogen, from ethanol *in vitro*, but no information exists regarding whether the ACH production depends on oral microbiota profiles.

**Objective and Design:** To explore the salivary microbiota profiles with respect to ACH production ability in the oral cavity using a cross-sectional design.

**Results:** Using 16S rRNA gene amplicon sequencing, we classified 100 saliva samples into two types of communities (I and II). Salivary ACH production ability from ethanol was measured using gas chromatography and was found to vary over a 30-fold range. ACH production ability was significantly higher in the type I community, wherein the relative abundance of *Neisseria* species was significantly lower. Multivariate logistic regression analysis showed that the subjects with the type I community exhibited significantly higher probability of high ACH production ability than those with the type II community (*P* = 0.014). Moreover, the relative abundance of *Neisseria* species was inversely correlated with the ACH production ability (*P* = 0.002).

**Conclusion**: The salivary microbiota profile with a lower relative abundance of *Neisseria* species was independently associated with high ACH production ability, despite *Neisseria* species are dominant producers of ACH *in vitro*.

## Introduction

Acetaldehyde (ACH), the first metabolite produced during ethanol metabolism, is a carcinogen found in the oral cavity [1]. Recently, ACH production associated with the consumption of alcoholic beverages has been reclassified as highly carcinogenic (Group 1) by the International Agency for Research on Cancer of the World Health Organization [2]. In particular, increased ACH levels in saliva have been associated with increased risk for upper aerodigestive tract cancer [3–5], which was the seventh most common cancer in Japan in 2011 with one million newly diagnosed cases annually worldwide [6,7]. Increased ACH levels in saliva after ethanol consumption are suggested to be due to oxidation of alcohol by the oral microbiota [8,9].

To elucidate the contribution of the oral microbiota to ACH production in the oral cavity after ethanol consumption, researchers evaluated the capacity of oral bacteria to produce ACH from ethanol [10–12]. It has been shown that *Neisseria* species are capable of producing extremely high amounts of ACH when cultured *in vitro* in a medium containing ethanol [9]. In addition, we previously analyzed ACH production ability by the prevalent bacteria in the salivary microbiota of orally healthy subjects and found that several bacterial species in the oral microbiota possessed the ability to produce ACH [13]. In particular, *Neisseria* species were confirmed to produce significant levels of ACH from ethanol *in vitro*, in agreement with the reported finding by Muto et al. [9]. However, there have been no studies correlating ACH production ability with the complexity of the oral microbiota profiles, which encompass more than 700 bacterial species, 35% of which have not yet been cultured in the laboratory [14]. Analysis by 16S rRNA gene sequencing has made it possible to more comprehensively profile the oral microbiota in detail. This study aimed to explore the salivary microbiota profiles of healthy adults, using 16S rRNA gene amplicon sequencing, with respect to ACH production ability in the oral cavity. We hypothesized that the salivary microbiota profile with a higher relative abundance of *Neisseria* species would show higher ACH production ability.

## Materials and methods

### Study participants

One hundred healthy men, aged 20 years or older, were recruited from the Japan Ground Self Defense Force in the Nagasaki prefecture from September to October 2015. None of the participants used antibiotics during the survey. Written informed consent was obtained from all participants. This study was approved by the ethics committee of the faculty of the Kyushu University (number 27–71). Data were collected, analyzed, and reported in accordance with the Strengthening the Reporting of Observational Studies in Epidemiology guidelines [15].

### Sample size

Sample size was calculated by statistical software (nQuery Advisor, Statistical Solutions, Saugus, MA), using the two-tailed hypothesis test, with a significance level of 5% and power of 80% with an allocation ratio of 1:1. To detect a statistically significant 30 percentage point difference in the ACH production ability based on the pilot trial data between the groups, a sample of 31 participants per group (62 in total) was needed.

### Oral examinations and questionnaires

All oral examinations were performed by the same dentist (S.Y.). Oral examination data included number of teeth, probing pocket depth (PPD), bleeding on probing (BOP), and plaque index score. PPD on the mesiobuccal and midbuccal sites for all teeth except the third molars was measured, and the mean PPD was recorded as a periodontal parameter. BOP was used as a measure of gingival inflammation [16]. Plaque index score was used as a measurement of oral hygiene status [17]. Examiner reliability for the oral examination was verified by intra-examiner calibration with volunteers, who had similar characteristics to the participants in the study; Cohen’s *κ* value >0.8 indicated high intra-examiner reliability. Additional information, including tooth brushing frequency, mouth rinse use, regular dental visits, frequency of alcohol intake, and smoking habit, was obtained using a self-administered questionnaire. Tooth brushing frequency was categorized as ≤2 times per day or ≥3 times per day. Mouth rinse use was assessed using the question, ‘Do you currently use mouth rinse?’ to which respondents answered ‘yes’ or ‘no’. With respect to regular visits to the dentist, the participants were categorized as those who did or did not visit the dentist for oral care at least once a year. Frequency of alcohol intake was classified as daily or non-daily drinker. Smoking habit was classified as current or non-current smoker.

### Saliva collection

Saliva sample collection was performed at 10:00 and 12:00 am before the oral examination. Participants were instructed to refrain from eating, drinking, or brushing their teeth at least 1 h prior to sampling. During sampling, participants were instructed to sit in a chair and chew gum free of sweeteners, food additives, and flavoring agents for 3 min, and stimulated saliva samples were collected in sterile plastic tubes, as previously described [18]. The saliva samples were stored at −80°C until analysis.

### Method for measuring acetaldehyde production ability

Measurement of ACH production ability was carried out, according to the previously described method with minor modifications [12]. The saliva sample (400 μL) was thawed, thoroughly mixed, and transferred into a gas chromatograph vial. Thereafter, 50 μL of 110 mM ethanol (final concentration 11 mM) was added and the vial was immediately sealed tightly. The sealed sample was incubated at 37°C for 30 min. The reaction was stopped by adding 50 μL of 6 M perchloric acid (PCA) via the polytetrafluoroethylene/silicon diaphragm of the vial. The ACH concentration in 5 mL of the vial headspace gas was determined using the sensor gas chromatograph SGEA-P2 (FIS Inc., Hyogo, Japan). To measure artefactual ACH, 50 μL of PCA was first added to the saliva sample (400 μL), followed by ethanol. The ACH concentration of this control sample was subtracted from that of the test sample. The ACH production ability in saliva (ppb/mL) was defined to be the net increment in the ACH concentration in the vial headspace per 1 mL of saliva in 30 min.

### Bacterial quantification via real-time PCR

DNA was extracted from each saliva sample using a bead-beating method, as previously described [19]. Total bacterial count per mL saliva was assessed with a QuantiFast SYBR Green PCR kit (Qiagen, Hilden, Germany) and a StepOne Real-Time PCR System (Applied Biosystems) with the universal bacterial primers 806F (5′-TTAGATACCCYGGTAGTCC-3′) and 926R (5′-CCGTCAATTYCTTTGAGTTT-3′), as previously described [20]. The *Porphyromonas pasteri* 16S rRNA gene, which was inserted into the pBluescript SK II (+) vector plasmid (Stratagene, La Jolla, CA), was used as the real-time control.

### 16S rRNA gene amplicon sequencing

Barcoded pyrosequencing analyses of the 16S rRNA gene were performed using Ion PGM (Thermo Fisher Scientific, MA), a next-generation sequencer, as previously described [21]. In brief, the V1–V2 regions of the 16S rRNA gene in each saliva sample were amplified using the following primers: 8F (5′-AGAGTTTGATYMTGGCTCAG-3′) with the Ion Torrent adaptor A and a sample-specific 8-base tag sequence, and 338R (5′-TGCTGCCTCCCGTAGGAGT-3′) with the Ion Torrent adaptor trP1 sequence. Following PCR amplification, purification, and quantification, equal amounts of the purified PCR amplicon products were pooled and gel-purified. Emulsion PCR and enrichment of template-positive particles were performed using an Ion PGM Template OT2 400 Kit (Thermo Fisher Scientific) in the Ion One Touch 2 system (Thermo Fisher Scientific). The enriched particle was loaded onto an Ion 318 v2 chip (Thermo Fisher Scientific) and sequencing was performed on the Ion PGM (Thermo Fisher Scientific) using an Ion PGM Hi-Q Sequencing kit (Thermo Fisher Scientific).

Low-quality reads were excluded using a script written in R (version 3.1.1), according to the following criteria: read length of ≤200 bases (not including the tag sequence), average quality score ≤25, correct forward primer sequence not included, correct reverse primer sequence (one mismatch allowed) not included, or the presence of a homopolymer run >6 nt. The quality-checked reads were assigned to their corresponding sample by examining the tag sequence. After removal of primer and tag sequences, similar sequences were assigned to operational taxonomic units (OTUs) using UPARSE [22], with a minimum pairwise identity of 97%. The taxonomic classification of the representative sequences was determined by performing a BLAST search against 831 oral bacterial 16S rRNA gene sequences (HOMD 16S rRNA RefSeq version 14.51) from the Human Oral Microbiome Database [23]. Nearest-neighbor species with ≥98.5% identity were selected as candidates for each representative OTU. The taxonomic classification of the sequences without a hit was determined using the Ribosome Database Project (RDP) classifier with a minimum support threshold of 80%. The obtained sequence data were deposited in the DDBJ Sequence Read Archive (DRA006849).

### Community type analysis

Bacterial community types were identified, as described previously [24]. The saliva samples were clustered based on the relative abundance of genera using the Jensen-Shannon divergence distance metric, and the partitioning around medoids (PAM) clustering algorithm with the pam function in the cluster library of R. The number of clusters was chosen by maximizing the Calinski–Harabasz (CH) index. The permutational multivariate analysis of variance (PERMANOVA) was performed using the adonis function in the vegan library of R. The most discriminant OTUs were determined using the linear discriminant analysis effect size (LEfSe) method [25]. The linear discriminant analysis score (LDA score) was shown to indicate the effect size of each OTU.

### Statistical analysis

The participants (*n* = 100) were classified into two groups designated as high ACH production ability (greater than third quartile) and non-high ACH production ability (less than third quartile), based on the magnitude of salivary ACH production ability. Characteristic differences between the participants in the high and non-high ACH production ability groups were determined using the Pearson’s Chi-square test for categorical variables and the Mann–Whitney *U* test for continuous variables. To examine the association between bacterial community types and salivary ACH production ability, we estimated the odds ratios (ORs) and 95% confidence intervals (CIs) for high ACH production ability based on bacterial community types using logistic regression models. A multivariate logistic regression model was created to adjust for possible confounding factors in the association between bacterial community types and salivary ACH production ability. The multivariate analysis included all independent variables that were significantly associated with salivary ACH production ability at a significance of *P*-value <0.05 in the descriptive analysis (Table 2). Additionally, a correlation between the most discriminant OTU in each bacterial community type and salivary ACH production ability was evaluated, using the Pearson’s correlation coefficient. All statistical analyses were performed using R or IBM SPSS statistical software (version 24; IBM Corp., Armonk, NY) and two-sided *P*-values <0.05 were considered statistically significant in all cases.

**Table 1.** Mean relative abundances of bacterial genera in each community type.

	Relative abundance (%, Mean ± SD)		
	Type I (*n* = 65)	Type II (*n* = 35)	*P*-value
*Streptococcus*	43.1 ± 10.8	30.3 ± 9.7	<0.001
*Rothia*	18.4 ± 9.8	12.2 ± 6.8	0.001
*Prevotella*	6.4 ± 4.1	4.2 ± 3.1	0.002
*Actinomyces*	9.2 ± 7.7	6.8 ± 4.2	0.126
*Veillonella*	4.4 ± 2.6	3.3 ± 1.7	0.122
*Granulicatella*	2.9 ± 2.0	2.7 ± 1.2	0.914
*Leptotrichia*	1.3 ± 1.6	1.0 ± 1.3	0.418
*Lautropia*	1.2 ± 2.8	1.5 ± 2.0	0.024
*Gemella*	1.6 ± 1.2	2.7 ± 1.7	<0.001
*Haemophilus*	1.5 ± 1.5	3.2 ± 2.6	<0.001
*Porphyromonas*	1.3 ± 1.7	4.9 ± 4.1	<0.001
*Fusobacterium*	0.9 ± 0.8	5.0 ± 3.6	<0.001
*Neisseria*	3.9 ± 4.3	16.2 ± 7.7	<0.001

SD, standard deviation. Only 13 genera with a mean relative abundance of ≥1% within each type are shown.

**Table 2.** Characteristics of participants according to acetaldehyde production ability.

	Acetaldehyde production ability		
	High (*n* = 25)	Non-high (*n* = 75)	*P*-value
Age (years)	31.0 (24.0–45.0)	30.0 (20.0–50.0)	0.576
Number of present teeth	29.0 (27.0–32.0)	29.0 (23.0–32.0)	0.878
Plaque score	0.3 (0.0–1.7)	0.3 (0.0–1.8)	0.769
Bleeding on probing (%)	7.1 (0.0–59.3)	10.7 (0.0–92.3)	0.271
Probing pocket depth (mm)	1.5 (1.2–4.1)	1.6 (1.0–4.1)	0.155
Total bacteria in saliva (log_10_ copies/ml)	8.5 (7.8–9.2)	8.2 (7.5–9.3)	<0.001
Bacterial community types (%)			0.007
Type I	88.0	57.3	
Type II	12.0	42.7	
Tooth brushing frequency (%)			0.463
≤2 times per day	60.0	69.3	
≥3 times per day	40.0	30.7	
Mouth rinse use (%)			0.388
Yes	12.0	21.3	
No	88.0	78.7	
Regular visits to the dentist (%)			0.488
Dental care at least once every year	64.0	54.7	
No dental care at least once every year	36.0	45.3	
Frequency of alcohol intake (%)			0.047
Every day	36.0	16.0	
Non-every day	64.0	84.0	
Smoking habit (%)			0.645
Current smoker	56.0	48.0	
Non-current smoker	44.0	52.0	

Continuous variable expressed as median (range); categorical variables, as percentage.

## Results

**Figure 1.**
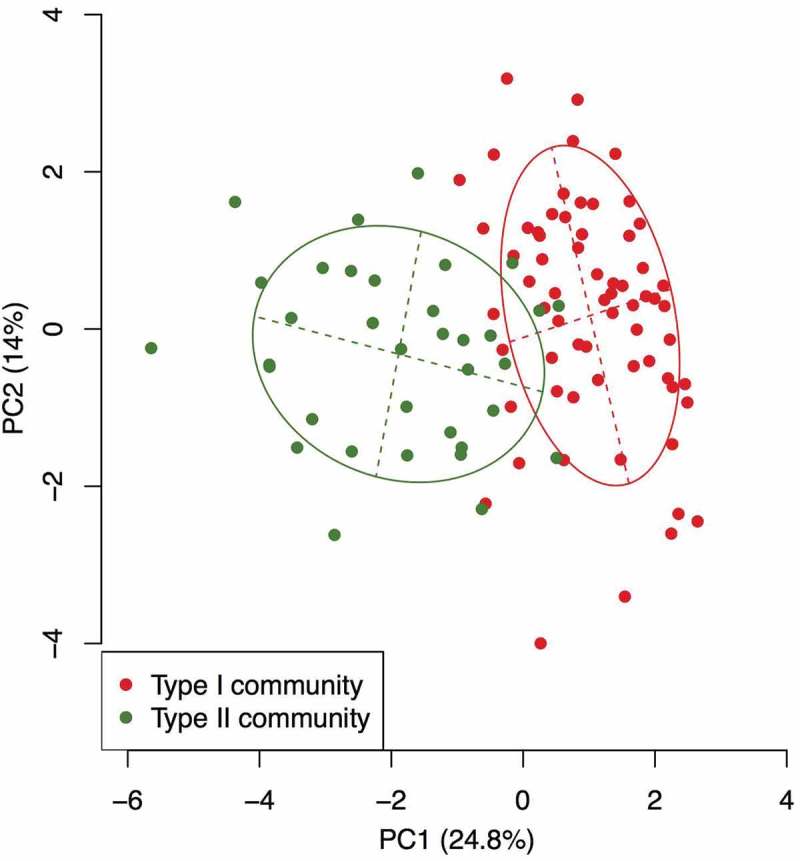
Principal component analysis showing similarity of bacterial compositions of saliva samples from each subject. The bacterial compositions belonging to each type are depicted using different colors. These two components explain the 38.8% variance. The intersection of the broken lines indicates the center of gravity for each type. The ellipse covers 67% of the samples belonging to each type.

**Figure 2.**
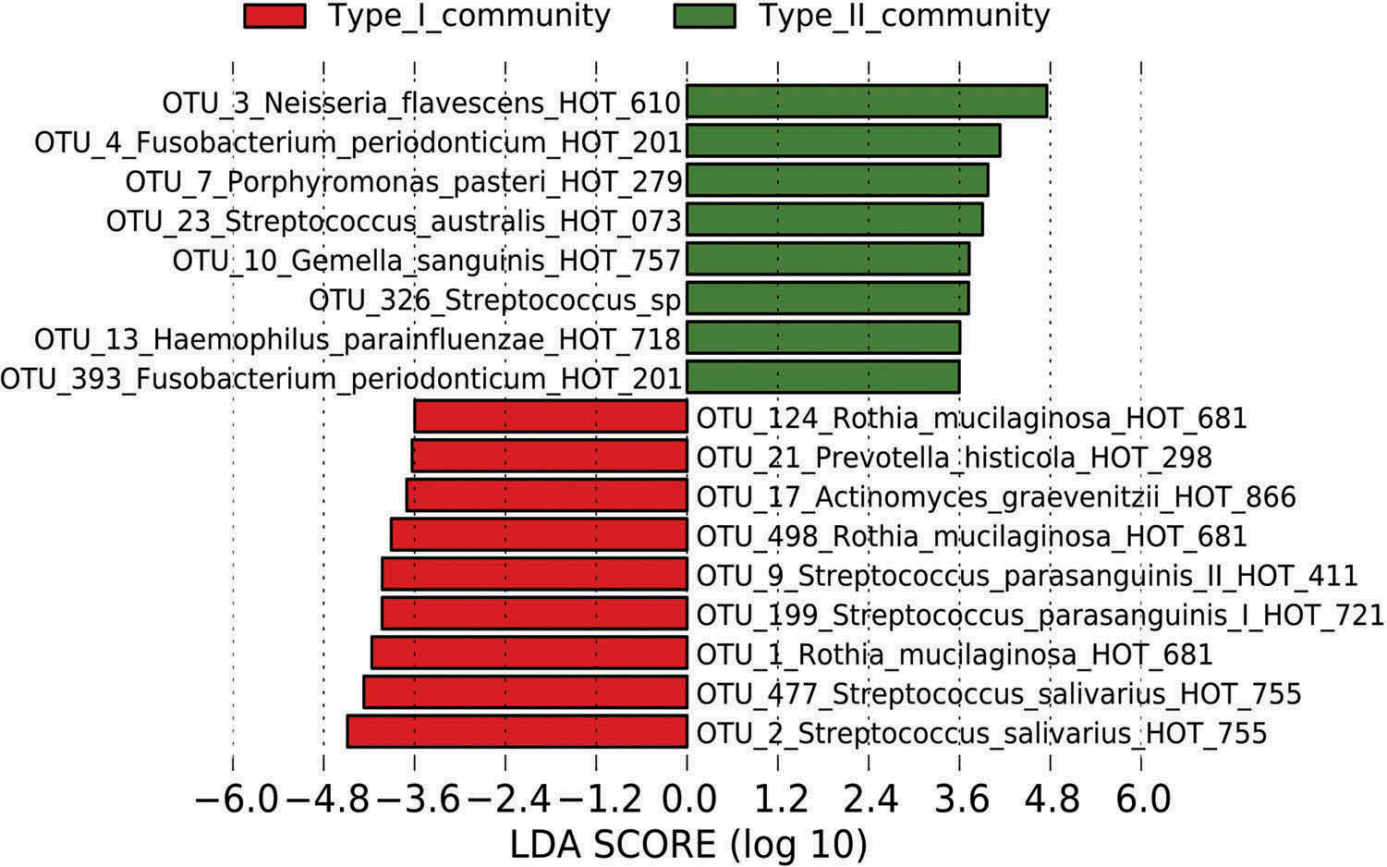
Most differentially abundant OTUs between types I and II microbiota. Each bar plot representing the most differentially abundant OTUs between the community types I and II as detected by a LDA effect size (LEfSe) analysis (LDA score >3.5). OTUs signature specific to the community types I and II are, respectively, in red and green.

We classified the bacterial communities of the saliva samples as either type I (*n* = 65) or type II (*n* = 35) using PAM clustering (PERMANOVA *R*^2^ = 0.21, *P*-value <0.001) and visualized the difference between the two types using principal component analysis (Figure 1). The type I community was dominated by *Streptococcus* and *Rothia* species (Table 1), and OTUs corresponding to *Streptococcus salivarius* HOT-755 and *Rothia mucilaginosa* HOT-681 were identified as the most differentially abundant OTUs by the LEfSe approach to detect discriminant OTUs (Figure 2). In contrast, the type II community was dominated by *Neisseria, Fusobacterium*, and *Porphyromonas* species (Table 1), and OTUs corresponding to *Neisseria flavescens* HOT-610, *Fusobacterium periodonticum* HOT-201, and *Porphyromonas pasteri* HOT-279 were identified as the most differentially abundant OTUs (Figure 2). In particular, the relative abundance of *Neisseria* species was significantly lower in the type I community (3.9 ± 4.3% in type I vs 16.2 ± 7.7% in type II).

**Figure 3.**
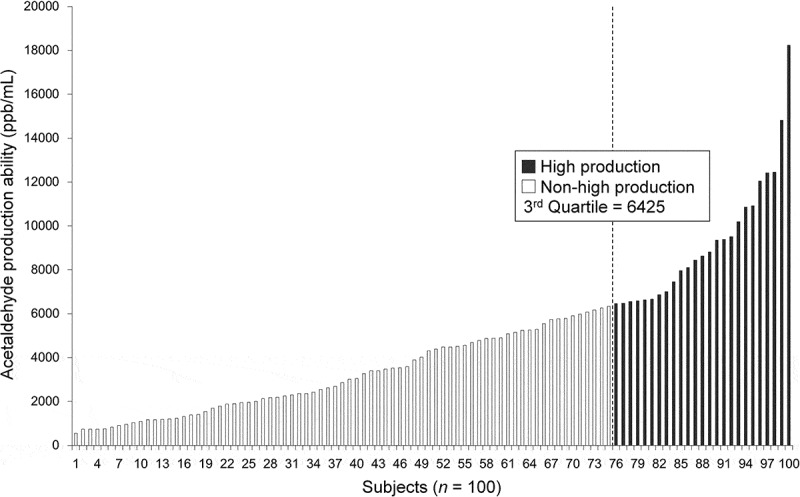
Distribution of salivary acetaldehyde production ability with 11 mM ethanol concentration. The data represent the means of three independent experiments.

The distribution of salivary ACH production ability (ppb/mL) is shown in Figure 3 (median, 4,346; range, 547–18,231). The differences in the characteristics of participants between high ACH production ability group (*n* = 25; median age and range 31 and 24–45 years) and non-high ACH production ability group (*n* = 75; median age and range 30 and 20–50 years) are shown in Table 2. The prevalence of the type I community was significantly higher in the high ACH production ability group compared to that in the non-high ACH production ability group. From a different perspective, the ACH production ability was significantly higher in the type I community (5,508 ± 3,658 ppb/mL in type I vs 3,180 ± 2,223 ppb/mL in type II). Total bacterial count in the saliva of the members of the high ACH production ability group was significantly higher than that in the members of the non-high ACH production ability group. With respect to frequency of alcohol intake, the difference in the bacterial community prevalence between daily and non-daily drinkers in the high ACH production ability group was shown to be statistically significant. In contrast, there were no significant differences with respect to age, number of present teeth, PPD, BOP, plaque index score, tooth brushing frequency, mouth rinse use, regular visits to the dentist, and smoking habits between the two groups.

The results of multivariate logistic regression analysis for the association between microbiota types and high ACH production ability are shown in Table 3. After adjusting for total bacterial count in the saliva and frequency of alcohol intake, the subjects with the type I community exhibited significantly greater odds for high ACH production ability (OR = 5.34, 95% CI = 1.40–20.29) than those with the type II community.

**Table 3.** Multivariable analysis of the association between community types and high acetaldehyde production ability.

Bacterial community types	Adjusted OR^a^ (95% CI)	*P*-value
Type II (*n* = 35)	1.00 (reference)	
Type I (*n* = 65)	5.34 (1.40–20.29)	0.014

OR, odds ratio; CI, confidence interval. ^a^Adjusted for total bacterial count in saliva and frequency of alcohol intake.

**Figure 4.**
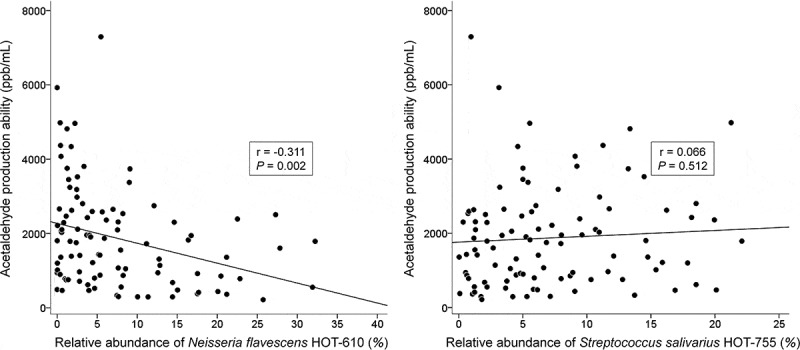
Correlations of *Neisseria flavescens* HOT-610 and *Streptococcus salivarius* HOT-755 with salivary acetaldehyde production ability.

The association between the most discriminant OTU in each community type and the salivary ACH production ability is shown in Figure 4. A statistically significant inverse correlation was observed between the abundance of *N. flavescens* HOT-610 and ACH production ability (*r* = −0.311; *P*-value = 0.002), but no such correlation was observed between the abundance of *S. salivarius* HOT-755 and ACH production ability (*r *= 0.066; *P*-value = 0.512).

## Discussion

In this study, we demonstrated that the ACH production ability was significantly different in two bacterial community types derived from saliva samples from healthy adults, indicating that the subjects with type I community, characterized by species such as *S. salivarius* and *R. mucilaginosa*, showed higher ACH production ability than those of the type II community, characterized by species such as *N. flavescens* and *F. periodonticum*. These findings highlighted that the salivary microbiota with higher relative abundance for *Neisseria* species independently associated with lower ACH production ability, although *Neisseria* are oral bacterial species that are known high-level ACH producers [9,13]. Furthermore, the association was independent of the total salivary bacterial count, alcohol intake frequency, and other important health characteristics. To our knowledge, this is the first population-based sample study to elucidate the oral microbiota profiles affecting the ACH production ability using next-generation sequencing.

*Neisseria* species, present in the saliva of ≥80% of the subjects in this study, have been reported to produce considerable amounts of ACH from ethanol *in vitro* [9,13]. However, the salivary microbiota with less relative abundance of *Neisseria* species (type I community) showed significantly higher ACH production ability than that with the higher relative abundance of *Neisseria* species (type II community). In addition, a significant inverse correlation between the relative abundance of *Neisseria* species and the ACH production ability in saliva was shown in this study. These findings suggest that an *in vivo* oral environment-derived type II community may arrest ACH production by *Neisseria* species. For instance, there is a possibility that some of the predominant metabolites produced by the type II community suppress the expression of the alcohol dehydrogenase gene of *Neisseria* species. Transcriptomic analysis in the future may be helpful in testing this hypothesis.

Alternatively, the expression levels of the alcohol dehydrogenase gene may be considerably higher in the type I community than those in the type II community. Many species of bacteria capable of producing relatively high amounts of ACH, such as *S. salivarius, R. mucilaginosa*, and *Prevotella histicola* [13], were observed in relatively higher proportions in the type I community, even though the relative abundance of *Neisseria* species was less. Our previous study confirmed that *S. salivarius, R. mucilaginosa*, and *P. histicola* are the predominant members of the salivary microbiota even in orally healthy subjects and are capable of producing relatively high amounts of ACH from ethanol *in vitro* [13]. Although these species do not produce as much ACH as *Neisseria* species, it is possible that their sheer abundance compensates for the absence of *Neisseria* and explains the high levels of ACH produced in subjects with the type I microbiota. Further studies, using metagenomic approaches, are required to elucidate the functional differences in ethanol metabolism between the type I and type II communities.

Our previous population-based studies also classified the salivary bacterial communities into two types in a manner similar to that described in this study. The subjects that resembled those with the type II community in this study showed better oral health conditions (i.e. had fewer teeth with dental caries, were non-smokers, and other such factors) and had a lower risk for pneumonia-related death [20,26], suggesting that the predominance of *N. flavescens, F. periodonticum*, and *P. pasteri* is an indicator of healthy oral microbiota. Furthermore, a recent report by Zaura et al. suggested that a bacterial community type characterized by a predominance of *N. flavescens* and *Neisseria subflava* positioned away from dysbiosis [27]. However, it is unexpected that a salivary microbiota predominant with *Neisseria* species is beneficial for health despite their capacity for high ACH production ability. In contrast, in this study, we demonstrated that the type II community dominated by *N. flavescens* showed significantly lower ACH production ability, showing an inverse correlation with the relative abundance of *Neisseria* species in the microbiota. Although the reason for this contradictory finding is still not known, our data are relevant to and highlight the clinical significance of the comprehensive analyses of the microbiota.

Till date, characterization of isolated bacteria is indispensable to understanding the virulence of pathogens. Our study suggests that the characteristics of bacteria *in vitro* do not necessarily reflect their behavior when present in habitats, such as the oral microbiota. Contrary to our hypothesis, the ACH production ability showed an inverse correlation with the relative abundance of *Neisseria* species, which are known ACH producers among oral microbes. There are complex interactions within the microbiota, which result in the expression of unexpected phenotypes by the community. Further comprehensive analyses are important to elucidate the true virulence of the microbiota.

Although ACH is a carcinogen in the oral cavity, all our study participants were healthy adults and cannot be attributed with carcinogenic oral ACH levels. In contrast, we confirmed that the ACH production ability by the salivary microbiota among healthy adults varied over a 30-fold range. This broad range guarantees objectivity in evaluating the association between the bacterial composition of the microbiota and the ACH production ability.

With regard to the effect of freezing and thawing of saliva samples, we preliminarily compared the ACH producing activity of pre- and post-freezing saliva samples from healthy adults (including the man who had high percent relative abundance of *Neisseria* species in the salivary microbiota): pre-freezing samples were fresh saliva immediately after collection; post-freezing samples were samples thawed after 1 week in frozen condition. As a result of the preliminary data, there was no statistical difference in the ACH producing activity between pre- and post-freezing samples (*P*-value = 0.140). Thus, there is little probability that *Neisseria* species survived freezing and thawing better than other species.

In conclusion, a noteworthy feature of this study was the marked difference in the salivary ACH production ability, depending on the oral microbiota of healthy adults. Since the relative abundance of *Neisseria* species in the salivary microbiota was negatively correlated with the ACH production ability, these data emphasize that the salivary microbial profile is a key determinant of ACH production, which can occur independent of the presence of *Neisseria* species.
